# Preparedness and Readiness Strategies for Addressing the COVID-19 Pandemic in Fragile and Conflict Settings: Experiences of the Gaza Strip

**DOI:** 10.3389/fpubh.2021.766103

**Published:** 2021-11-22

**Authors:** Samer Abuzerr, Said Abu-Aita, Ismail Al-Najjar, Azzam Abuhabib, Heba Al-Jourany, Kate Zinszer

**Affiliations:** ^1^Department of Social and Preventive Medicine, School of Public Health, University of Montreal, Montréal, QC, Canada; ^2^Quality Improvement and Infection Control Unit, Ministry of Health, Gaza, Palestine; ^3^Crisis Management Consultant, Gaza, Palestine; ^4^Disaster and Crisis Management Master Programme, Islamic University of Gaza, Gaza, Palestine; ^5^Water Technology Ph.D. Joint Programme, Islamic University of Gaza, Gaza, Palestine; ^6^Disaster Risk Reduction Researcher, Gaza, Palestine; ^7^Department of Social and Preventive Medicine, School of Public Health, University of Montreal, Montréal, QC, Canada

**Keywords:** preparedness, readiness, strategies, fragile settings, COVID-19, Gaza Strip

## Abstract

The COVID-19 pandemic is a global public health threat of serious concern, especially in conflict settings that face fragility and lack adequate resources and capacities. Gaza suffers from a blockade imposed by the Israeli occupation, environmental deterioration, confiscation of lands, demolition of houses and hospitals, restrictions on movement, lack of control over natural resources, and financial constraints. Gaza's population is consequently living in a poor humanitarian situation with high unemployment rates, poverty, over-crowdedness, and a weak health system. This makes Gaza incredibly fragile and affects its ability to respond to the COVID-19 pandemic effectively. The pandemic is expected to deepen Gaza's systems' fragility, which is already overstretched beyond their limits. This will hinder its capacity to deal with the pandemic, and other pre-existing pressing humanitarian needs. Therefore, in this review, we comprehensively explored Gaza's policy failures and successes related to the COVID-19 preparedness and response by state and non-state actors and recommend potential solutions and alternatives. We have addressed critical issues including the health system, water, sanitation, hygiene, socio-economic, education, food security, and others. In Gaza, effectiveness in combating the COVID-19 pandemic can only come from committed political will, transparency from all regulators, strategic dialogue, comprehensive planning, and active international support.

## Introduction

The current reactive response of local authorities in Gaza has been effective in combating COVID-19. The number of cases and fatalities in the first 6 months of the crisis was relatively low compared to numbers in neighboring regions, including in the West Bank and Egypt. This has been possible not only because all incoming travelers to Gaza via Israel and Egypt enter a 21-day mandatory quarantine in designated facilities but also because entry and exit into Gaza are extremely limited due to the blockade (1).

**Figure 1:**
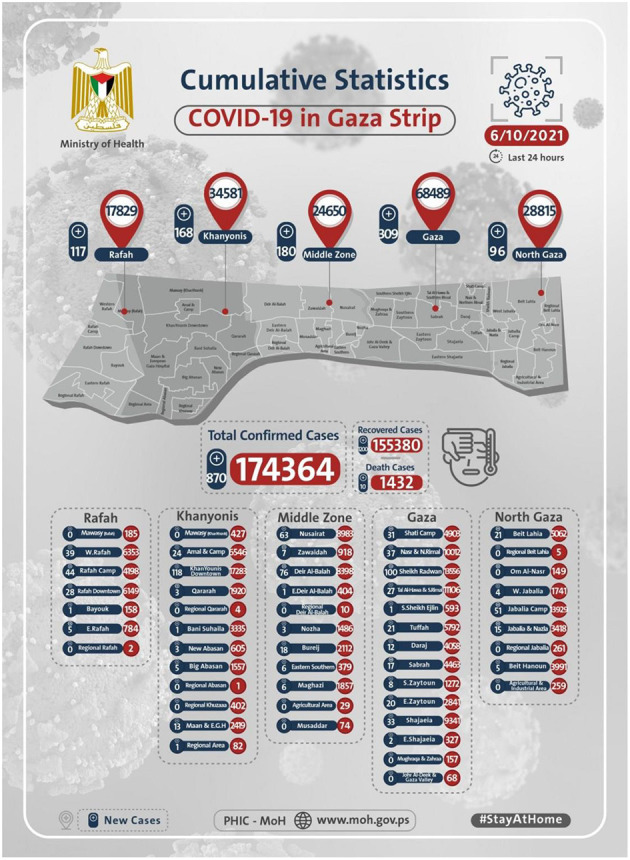
The total number of COVID-19 cases in the Gaza Strip, including the cases' distribution on its five governorates. Source: http://www.moh.gov.ps/portal/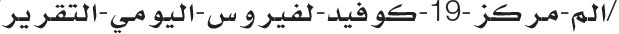.

Until 24 August 2020, the total number of infections from inside quarantine centers for arrivals to Gaza stood at 109, including one death (2). On that date, the situations have been turned upside down, as four persons residing in Al Maghazi camp were diagnosed with positive COVID-19. As a response to confronting the spread of COVID-19 in the community, the local authorities have imposed complete isolation on the Al Maghazi camp, besides imposing a complete curfew in all Gaza Strip (GS) governorates for 48-h extended several times. The number of cases increased significantly, and on the date of writing these lines, 6 October 2021, it reached 174364 COVID-19 cases, including 1,432 deaths (3). The map in Figure 1 herein shows the total number of COVID-19 cases in the GS, including the cases' distribution on its five governorates.

Therefore, the COVID-19 is expected to excavate Gaza's Health System's (HS) fragility, which is already overloaded beyond its limits. The Ministry of Health (MOH) declared a state of emergency by allocating all efforts and available medical supplies in coping with the pandemic (4–6).

Therefore, in this review, we comprehensively explored Gaza's policy failures and successes related to the COVID-19 preparedness and response by state and non-state actors and recommend potential solutions and alternatives. We have addressed critical issues, including the health system, water, sanitation, hygiene, socio-economic, education, food security, and others. To collect data to help in objectives fulfillment, we have done an extensive reviewing and referring to local institutional websites, reports, factsheets, and other national, regional, and international English and Arabic documents, statistics, and studies released by unions and representative groups, World Health Organization (WHO), United Nations Office for the Coordination of Humanitarian Affairs (OCHA), clusters, Palestinian Ministries. In addition to several academic databases and search engines with no limit to the time of publication.

## The Local Preparedness and Readiness Strategies for Addressing the Pandemic

According to the COVID-19 adaptation strategy pointed out by WHO, six critical criteria are to be met. The following points state these six criteria as benchmarking drivers to compare local measures relevancy and comply as well as to identify gaps (7):

– Transmission control to a manageable level by the HS is determined by the capacity to reserve substantial clinical care capacity. This would be achieved by maintaining all sporadic and cluster cases newly or already discovered. Practically, local authorities took some exceptional testing measures back on 22 March 2020 when the first cases were detected on Rafah crossing point for two travelers arriving from Egypt.
– The measurements consisted of conducting Polymerase Chain Reaction (PCR) tests for all returnees coming from Egypt and Israel as well as imposing compulsory quarantine for 21-days. More than 30 quarantine centers, including schools, hotels, primary health care centers, hospitals, and newly established centers, were prepared to receive and accommodate Gazans who are returning from abroad (4).
– Borders were fully closed and occasionally opened for the returnees for few days between March and August 2020 while PCR tests and compulsory quarantine continued accordingly. When the first case was detected inside the community on 24 August 2020, a full lockdown was imposed. PCR tests were performed for suspected contacts and compulsory quarantine for both infected patients and contacts. With the increased number of cases detected, the authorities shifted to mandatory home quarantine for contacts and suspected cases. In contrast, treating confirmed cases received treatment and quarantined at the European hospital to the south of the GS^1^.
– Sufficient HSs and public health capacities are in places that significantly assist in moving from detecting and treating severe cases toward detecting and isolate all infected patients regardless of severity and origin. This can be achieved by detecting suspicious cases, testing all suspected cases, isolating confirmed cases, and closing quarantine contacts for 14 days. The exceptional measures responding to COVID-19 made by the MOH are represented by several measures including but not limited to (1) Quarantines establishment and supervision for returnees as well as conducting PCR tests for all returnees and contacts for the period from March to August 2020. (2) Full dedication of European hospital in the Khan Younis area, to the south of GS, for COVID-19 cases treatment. (3) After discovering infected cases inside the community by 24 August 2020, the MOH recommended a full lockdown imposed by security forces to prevent widespread pandemics. (4) Exceptional measures and protocols were announced and followed by medical staff at all hospitals, as well as 14 days of compulsory quarantine for medical staff dealing with COVID-19 cases. (5) Daily updates announcing COVID-19 figures are held.
– Minimizing outbreak risks in high-vulnerability settings by identifying major drivers and/or amplifiers of COVID-19 transmission as well as having appropriate minimization measures of new outbreaks and nosocomial transmission. Following the discovery of infected cases within the community on 24 August 2020, a full lockdown was imposed. In addition, a restricted curfew from 8:00 pm to 7:00 am daily is effectively announced as well as complete prevention of movement for people <16 years old and above 60 years toward minimizing outbreaks and reduce risks for vulnerable groups in addition to full prevention of movement between governorates except with permission in all times. All mosques, gathering/wedding halls, weekly markets, chalets and resorts, seaside and beach, shopping malls, and any other related activity involving gathering are entirely restricted and prohibited.
– Establish preventive measures at the workplace to reduce associated risks including, social distancing, hand washing, respiratory etiquette, and potentially temperature monitoring. Following the discovery of infected cases within the community on 24 August, 2020, preventive measures were strictly imposed, including face mask-wearing, sanitizing materials provision at all shops, pharmacies, and others. Those not complying with such measures may face fines and penalties imposed by local authorities and security forces. To date, all public services staff except those involved in emergency activities related to COVID-19 response (e.g., medical, security, and municipal staff) as well as educational institutes, non-governmental organizations (NGOs), and other businesses are under lockdown under full suspension and/or working from home mode. It was clearly announced by local authorities to commit to using preventive measures at all workplaces at all times.
– Full engagement of communities and promoting the understanding of the need to shift from detecting and treating severe cases to detecting and isolating all COVID-19 cases. Behavioral prevention measures have to be maintained with crucial roles for all individuals to be played. Various awareness campaigns and activities were organized by local authorities, NGOs, and others to involve the public in committing to the lockdown measures as well as the use of preventive measures (facemasks, sanitizers, social distancing, and others). These activities and campaigns are actively running through social media, press, local radios, and other means trying to reach as many people as possible to reduce outbreaks risks. This is one side of community engagement that sounds more like engaging by providing good practice to deal with the pandemic. However, at the planning and response level, local authorities tend to be taking the lead with no room whatsoever for the community to involve or participate except for public compliance angle despite the great efforts made by the WHO and The United Nations Children's Fund (UNICEF) in this regard (8).

It is worth mentioning that public compliance with the abovementioned measures is observably low and tends to be deteriorating with time. This may have two sides: (1) level of ignorance related to the community attributed to the harsh situation associated with the long-lasting blockade and declined public services provided, leading to lack of trust between the community and local authorities (2) exhaustion and indolence by local authorities following up public compliance over time (9, 10).

According to a recently published study by Abuhabib and hiscolleagues, three potential risk scenarios associated with COVID-19 in Gaza were predicted and outlined. The three scenarios are: (1) Widespread of the virus with tens or hundreds of cases and fatalities daily with full control loss. (2) Cases continued to be deducted on the borders and quarantined without being spread within the community (3) Number of undiscovered cases is removed within the community leading to limited/unlimited infection inside the community (11).

The GS pandemic situation has experienced the shifting from the second scenario of having cases only at the borders to the third scenario of having infected cases within the community since 24 August 2020.

## The Humanitarian Crisis of the COVID-19 Pandemic in the Gaza Strip

The population density in GS of 5,453 persons/km^2^ is one of the highest worldwide. A population of 2 million inhabitants living in overcrowded camps. The socio-economic situation in Gaza has deteriorated throughout the 13 years of Israeli besiege. The intra-Palestinian division between the West Bank (WB) and the GS has adversely affected all life aspects, including national response, HS governance, financial and human resources, capacity and technologies, research, and coordination (12, 13).

### Socio-Economic Scene

The pandemic's repercussions are likely to profoundly impact socio-economic conditions in light of limited resources, possibly causing a collapse of the Strip. The Palestinian stagnated economy has been affected by the lockdown measures as people lost their jobs, and more are expected to become unemployed^2^. The Ministry of Labor (MoL) estimates that out of 130,000 registered workers, around 38,000 need urgent humanitarian assistance, which is exacerbating food insecurity and will, as a result, deteriorate their livelihood and health (14, 15). The unemployment and poverty levels are too high, reaching 51 and 53%, respectively (16).

According to the Palestinian Central Bureau of Statistics, a 6-month lockdown has declined the Gross Domestic Product (GDP) by 7.1% compared to 2019 and is expected to reach 14% by the end of 2020 (17). It is anticipated that the poverty level will get 64% with heavy losses that will incur by the owners of economic enterprises by the end of 2020^2^.

Following the onset of the pandemic, the production capacity of various industrial, commercial, tourist sectors witnessed a remarkable decline that could be attributable to the lack of purchasing power and citizens turning out to purchase essential foodstuffs, detergents, disinfectants, and personal protective equipment (PPEs) (18)^3^.

### Health System and Public Health Capacities

Despite the Geneva Convention requiring the occupying power to take “prophylactic and preventive measures necessary to combat the spread of contagious diseases and epidemics” (19). Israel's violations of human rights and international law have been increasing during the past few months of the COVID-19 crisis. The COVID-19 pandemic sheds light on the devastating impacts of the ongoing Israeli blockade since 2007 on the HS in Gaza, which impeded Palestinian efforts to combat COVID-19, including restricting the entry of essential materials needed for the health sectors such as equipment and medications, categorized as “dual-use” items, via the Israeli-controlled Karm Abu Salem crossing, the main commercial crossing. Accordingly, the COVID-19 pandemic sheds light on the devastating impacts of the ongoing blockade since 2007 on the HS in Gaza (20).

Healthcare services in GS are provided publicly by four primary providers at different levels: MOH through its governmental hospitals and primary healthcare clinics, United Nations for Relief and Working Agency (UNRWA), A UN agency providing primary healthcare to Palestine refugees in Gaza who are representing more than 70% of the population.^4^. Non-Governmental Organizations (NGOs), and finally, private hospitals and clinics. Practically, GS has 27 hospitals in total, 13 of which are governmentally managed by the Palestinian MOH with 1,500 beds capacity (21).

Meanwhile, Gaza's cancer patients (12,600 patients, 53% of them are females and 47% males) were trapped by politics and COVID-19 (22). Gaza's hospitals' ability to provide adequate diagnosis and treatment to cancer patients is severely limited due to chronic shortages of medicines and lack of medical equipment. Many patients need health care elsewhere in the occupied Palestinian territory or abroad. But to leave the GS for treatment, they must obtain a permit from Israeli authorities. The WHO estimates that around 1,200 patients need to leave Gaza for treatment (23). Moreover, the spike in COVID-19 cases in the occupied WB and Israel, as well as the 21-day quarantine imposed by the local authorities on patients returning to Gaza, has further put some people off applying for a permit in the first place (24).

### Educational Process

There are 695 kindergartens in the GS; 720 schools differ according to the supervisory authorities: 421 governmental schools, 274 UNRWA schools, and 25 private schools. Of them, 293 schools are working on a double shift system, morning and evening shifts. This reduces instructional hours on core subjects and foundation learning. In addition to overcrowded classrooms, there is limited time available to reinforce learning, support slow learners, and provide remedial education programs or extracurricular activities. Also, the number of university colleges is 17. These educational institutions are attended by more than half a million students (16, 25).

The Ministry of Education (MoE) in Gaza immediately launched its National Response Plan for COVID-19, including three scenarios to complete the educational process as follows (26):

Scenario 1: The ordinary full-time course and complete study plan with the usual school structure (average of 40 students per class).
Scenario 2: The educational process proceeds with an average of 20 students per class. The class students are divided into two groups, each attended by 3 days per week on a rotating basis.
Scenario 3: Complete lockdown of educational institutions.

In August 2020, after 5 months of closure, students had been smoothly returned and resumed classes they missed from the past year, with class sessions curtailed to four per day, recess canceled, and school canteens closed. Authorities were studying a full-fledged back-to-school start in September. This rare scene of normalcy came due to the non-detection of any COVID-19 cases outside quarantine centers.

Again, the COVID-19 pandemic overshadowed the education sector before the first month of school opening passed; on 24 August 2020, the Ministry of Education was pushed to apply the third scenario when the early four infections were detected among the population. Efforts to implement distance education have started immediately after schools' closure as an appropriate method for overcoming the current crisis and sustaining educational activities (27).

On 16 August 2021, the educational process resumed in a normal face-to-face mode for the first time since the pandemic took place for all education institutions across Gaza subjected to COVID-19 protocol announced by MoH (28).

### Water, Sanitation, and Hygiene

The containment measures of the COVID-19 pandemic have put an additional burden on WASH services providers. Since the pandemic onset, more water quantities are demanded to ensure hygiene practices at the household level and infection prevention control at healthcare facilities compared to the prior COVID-19 era (29, 30). The per capita daily water consumption is 88.3 liters per capita per day (lcd) in 2019, below the acceptable standard of 100 liters per capita per day recommended by the WHO (31, 32).

The chronic electricity deficit affecting the GS, alongside the longstanding shortage of adequate sanitation infrastructure, continues to result in the discharge of 100–108 million liters of untreated wastewater into the sea every day. These precarious nature facilities also generate a constant threat of sewage flooding in areas adjacent to reservoirs and pumping stations (33, 34).

It is estimated that WASH-related diseases accounted for over one-quarter of illnesses. The exposure to sewage collected around Gaza's neighborhoods is associated with an increased risk of acute diarrhea among children (35–38).

Gaza people's unaffordability to pay for improved WASH services and products, particularly during the COVID-19 pandemic, could also increase the likelihood of further spreading WASH-borne diseases in Gaza (16, 39, 40).

At the beginning of the COVID-19 crisis in Gaza, the solid waste collection has been suspended for 30% of households in Gaza since late April 2020 (41). That was due to municipalities' inability to pay the solid waste workforce's wages, which were already reduced previously. Municipalities were also forced to reduce funding allocated to purchase the fuel needed to run SWM vehicles and machinery in order to overcome funding shortage and keep services delivery even at minimum level despite the fact that some vehicles are old and require frequent maintenance (42). However, services had been restored to normal by August 2020. Consequently, this pandemic is expected to amplify the already overstretched Palestinian environment and HSs' fragility and make from Gaza the worst-case scenario concerning WASH-related diseases. It will hinder its capacity to deal with the other pre-existing needs of Gaza's population (43). With the pandemic spread, several measures were taken by local authorities as an attempt to reduce spreading, especially among staff and workers dealing with daily activities including waste collection and transport to landfills. Protective and prevention equipment PPEs (e.g., gloves, face masks, sanitizers) were distributed and used. New measures and standard operation procedures (SOPs) for handling quarantine centers garbage and wastes were also applied. For medical wastes at hospitals, special measures were also taken for departments dealing with COVID-19 patients in terms of waste collection and disposal only through incineration and not to send to landfills (42).

### Food Insecurity

Around 1.3 million people in Gaza suffer from severe or moderate food insecurity, according to the initial results of the latest socio-economic survey and food security in 2018 (44). The UNICEF and the World Food Program (WFP) reported that 23% of Gaza families have sub-optimal food consumption. Eighty percentage of these households receive some form of humanitarian aid (45, 46).

The livestock sector losses are likely to be catastrophic because of the inability to import, store or access fodder, with GS's agricultural inputs in need of $21 million (7 botanical products, 14 animal products).

The COVID-19 pandemic crisis has also caused Gaza's fishing sector damage, as this year's season is considered the worst in years. This is due to the closure of crossings and the cessation of various products abroad, including fish. The crisis has caused the price of fish in the GS to decline to about 50 and 70%, amid low demand, especially in light of the citizens' low purchasing power. About 4 thousand fishermen who support 24 thousand families work in the fishing profession in Gaza. The number of anglers has decreased significantly in recent years, while the fishing sector's total contribution has reduced to <1%.

The COVID-19 pandemic is likely to make matters worse. Unprecedented initial indicators, trends, and trends have arisen due to the epidemic, and the measures are taken to combat it and reduce its spread.

## Conclusions, Recommendations, and the Way Forward

As the world continues to bruise under the severe impact of COVID-19 with massive efforts and attempts toward developing the needed vaccine, already-vulnerable areas like GS continue to struggle and barely survive. The Israeli blockade and deepening internal dividing are severely hindering the efforts of COVID-19 spreading prevention. This fragile situation fed by a sequence of conflicts and escalations and worsened by COVID-19 is precarious and unstable. As comprehensively illustrated in this review, all sectors (Health, WASH, socio-economic, education, food security, and others) are in shaky shape with the possibility to collapse at any time, especially with the mandatory measures taken by the local authorities represented mainly by the lockdown and full closure. As such, sustaining preventive measures commitment by population and economy reviving with essential support to the HS is the foremost priority to avoid overall collapse.

Therefore, in this review, we have summarized and addressed critical issues affected by the COVID-19 pandemic, including the health system, water, sanitation, hygiene, socio-economic, education, food security, and others.

In general, several control measures are recommended to gradually adapt and live with COVID-19 in the long run with the overall situation under control and with a low level of infection and low mortality percentage as follows:

1. Support and empower the overall healthcare system and further engage UNRWA and international NGOs to provide PCR kits and medical supplies and increase the medical staff workforce using some temporary employment means.
2. Advocate and encourage population toward taking available vaccines and tremendously increase number of vaccinated people, as vaccinated individuals are far below required to face COVID- 19 widespread.
3. Maintain both regular and essential healthcare services using the newly established protective protocols.
4. Strategically increase the number of random PCR tests within the community to make numbers and figures more indicative and improve their accuracy and readiness. As recommended by the WHO, having a PCR positivity rate of 5% indicates that the number of cases is reduced significantly and might be considered guidance about testing screening level.
5. Gradually decrease the level of lockdown toward full lifting with the possibility of exceptionally lockdown certain areas with the high number of infected cases (specific indicators should be established to so with a clear link between the numbers of COVID-19 patients per 1,000 person).
6. Sustain the controlling measures that limit the people gathering and the number of persons presented per close and open areas (e.g., markets, barbershops, malls, bakeries, and others).
7. Imposing the public to wearing PPEs becomes a sort of daily practice for the overall population in the streets, markets, workplaces, and others.
8. Sustain the closure of mosques, wedding halls, weekly markets, and gathering places until further notice.
9. Adopt a new strategy for public transportation represented by committing to the use of PPEs by drivers and passengers and reducing passengers' number in public transportation modes.

## Author Contributions

IA-N, SA-A, HA-J, and KZ contributed on investigation, data curation, and writing. SA contributed on supervision and review & editing. All authors contributed to the article and approved the submitted version.

## Conflict of Interest

The authors declare that the research was conducted in the absence of any commercial or financial relationships that could be construed as a potential conflict of interest.

## Publisher's Note

All claims expressed in this article are solely those of the authors and do not necessarily represent those of their affiliated organizations, or those of the publisher, the editors and the reviewers. Any product that may be evaluated in this article, or claim that may be made by its manufacturer, is not guaranteed or endorsed by the publisher.

## Footnotes

^1^ https://moi.gov.ps/Home/Post/129944

^2^ https://www.worldbank.org/en/news/press-release/2020/06/01/palestinian-economy-struggles-as-coronavirus-inflicts-losses

^3^ http://www.fao.org/emergencies/results/en/?keywords=WEST%20BANK%20%26%20GAZA%20STRIP

^4^ https://www.unrwa.org/activity/health-gaza-strip

## References

[1] UNOCHA. Double Quarantine in Gaza: COVID-19 and the Blockade. (2020). Available online at: https://reliefweb.int/report/occupied-palestinian-territory/double-quarantine-gaza-covid-19-and-blockade (Retrieved April 18, 2021).

[2] UNOCHA. COVID-19 Emergency Situation Report 17. (2020). Available online at: https://www.ochaopt.org/content/covid-19-emergency-situation-report-17 (Retrieved June 17, 2021).

[3] Ministry of Health. (2021). Available online at: http://www.moh.gov.ps/portal/ 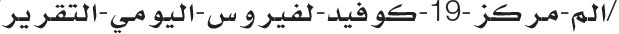

[4] Hammad J Tribe R. Social suffering and the psychological impact of structural violence and economic oppression in an ongoing conflict setting: the Gaza Strip. J Commun Psychol. (2020) 48:1791–810. 10.1002/jcop.22367 32399970

[5] WHO. Operational Considerations for Case Management of COVID-19 in Health Facility and Community: Interim Guidance. No. WHO/2019-nCoV/HCF_operations/2020.1. Geneva: World Health Organization (2020). Available online at: https://apps.who.int/iris/handle/10665/331492

[6] Abuzerr S Zinszer K Assan A. Implementation challenges of an integrated one health surveillance system in humanitarian settings: a qualitative study in palestine. SAGE Open Med. (2021) 9. 34504706 10.1177/20503121211043038 PMC8422815

[7] WHO. COVID-19 Strategy. (2020). Available online at: https://www.who.int/publications/i/item/covid-19-strategy-update-14-april-2020 (Retrieved April 12, 2021).

[8] Kaloti R. Situational Brief: Palestinian Refugees in the Occupied Palestine Territories During Covid-19. (2020).

[9] Hamad S Abu Hamra E Diab R Abu Hamad B Jones N Małachowska A. Exploring the Impacts of Covid-19 on Adolescents in the Gaza Strip. Gaza: Gender and Adolescence: Global Evidence (GAGE) | ODI (2020).

[10] Sen S. The Pandemic Under Siege: A View From the Gaza Strip. World Development (2020). p. 105063. 10.1016/j.worlddev.2020.105063 PMC7351377 32834376

[11] Abuhabib AA Abu-Aita SN Procter C Al-Smeri I. Unique situation of Gaza Strip dealing with COVID-19 crisis. Int J Infect Dis. (2020) 100:149–51. 10.1016/j.ijid.2020.08.070 32891733 PMC7470768

[12] UNCT. Gaza 10 Years Later. (2017). Available online at: https://unsco.unmissions.org/sites/default/files/gaza_10_years_later_-_11_july_2017.pdf (Retrieved March 1, 2021).

[13] AlKhaldi M Abuzerr S Obaid H Alnajjar G Alkhaldi A Khayyat A. Social determinants of health in fragile and conflict settings: the case of the gaza strip, palestine. In: Laher I editors. Handbook of Healthcare in the Arab World. Cham: Springer (2020). 10.1007/978-3-319-74365-3_203-2

[14] UNOCHA. COVID19 Emergency Situation Report 2. (2020). Available online at: https://www.ochaopt.org/sites/default/files/sitrep-2-30_march_2020.pdf (Retrieved April 13, 2021).

[15] Laher I. Handbook of Healthcare in the Arab World. Springer (2019).

[16] UNOCHA. Humanitarian Needs Overview. (2020). Available online at: https://www.ochaopt.org/sites/default/files/hno_2020-final.pdf (Retrieved March 17, 2021).

[17] PCBS. Economic Forecasts for the Year 2020, in Light of the Current Coronavirus Pandemic. (2020). Available online at: http://www.pcbs.gov.ps/site/512/default.aspx?tabID=512&lang=en&ItemID=3724&mid=3171&wversion=Staging (Retrieved February 17, 2021).

[18] Al-Monitor. Coronavirus Killing off What's Left of Gaza Strip's Economy. (2020). Available at: https://www.al-monitor.com/pulse/originals/2020/04/gaza-coronavirus-economy-tourism-unemployment-poverty.html (Retrieved April 27, 2021).

[19] ICRC. Geneva Convention Relative to the Protection of Civilian Persons in Time of War. 4th Geneva Convention (1949). Available online at: https://ihl-databases.icrc.org/applic/ihl/ihl.nsf/Treaty.xsp?action=openDocument&documentId=AE2D398352C5B028C12563CD002D6B5C

[20] Moss D Majadle G. Battling COVID-19 in the occupied palestinian territory. Lancet Global Health. (2020) 8:e1127–8. 10.1016/S2214-109X(20)30237-0 32827475 PMC7438081

[21] AA. Gaza Declares Lockdown Amid Community Spread of Virus. (2020). Available online at: https://www.aa.com.tr/en/middle-east/gaza-declares-lockdown-amid-community-spread-of-virus/1952137 (Retrieved April 1, 2021).

[22] WHO. Barriers for Patients in the Occupied Palestinian Territory. (2020). Available online at: http://www.emro.who.int/images/stories/palestine/documents/March_2020_Monthly.pdf?ua=1

[23] WHO. Gaza Patients' Painful Journey to Cancer Treatment. (2020). Available online at: http://www.emro.who.int/pse/palestine-news/gaza-patients-painful-journey-to-cancer-treatment.html

[24] Palestine UW. COVID-19 Quarantine Poses Added Challenges for Women With Cancer in Gaza. (2020). Available online at: https://palestine.unwomen.org/en/news-and-events/stories/2020/09/news-covid-19-poses-added-challenges-for-women-with-cancer-in-gaza (Retrieved March 10, 2021).

[25] UNOCHA. Education Undermined by the Deteriorating Humanitarian Situation in Gaza. (2018). Available online at: https://www.ochaopt.org/content/education-undermined-deteriorating-humanitarian-situation-gaza (Retrieved June 26, 2021).

[26] Palestine Authority P. State of Emergency, Palestine's COVID-19 Response Plan. (2020). Available online at: http://www.emro.who.int/images/stories/palestine/documents/Palestine_Authority_COVID-19_Response_Plan_Final_26_3_2020.pdf?ua=1 (Retrieved February 12, 2021).

[27] Al-Monitor. Gaza Students Embrace E-Learning to Avoid Coronavirus. (2020). Available online at: https://www.al-monitor.com/pulse/originals/2020/03/gaza-schools-move-to-e-learning-in-lockdown-due-to-covid-19.html (Retrieved March 17, 2021).

[28] OECD. COVID-19 Crisis Response in MENA Countries Report 2. (2020). Available online at: http://www.oecd.org/coronavirus/policy-responses/covid-19-crisis-response-in-mena-countries-4b366396/ (Retrieved March 22, 2021).

[29] UNOCHA. COVID-19 Emergency Situation Report 5. (2020). Available online at: https://www.ochaopt.org/content/covid-19-emergency-situation-report-5 (Retrieved April 28, 2021).

[30] WHO. Water, Sanitation, Hygiene, and Waste Management for SARS-CoV-2, the Virus That Causes COVID-19. (2020). Available online at: https://www.who.int/publications/i/item/water-sanitation-hygiene-and-waste-management-for-the-covid-19-virus-interim-guidance (Retrieved February 11, 2021).

[31] WHO. Domestic Water Quantity, Service, Level, and Health. (2003). Available online at: https://www.who.int/water_sanitation_health/diseases/WSH0302.pdf (Retrieved March 20, 2021).

[32] Shatat M Arakelyan K Shatat O Forster T Mushtaha A Riffat S. Low Volume Water Desalination in the Gaza Strip–Al Salam Small Scale RO Water Desalination Plant Case Study. Future Cities and Environment (2018). 4:11. 10.5334/fce.40

[33] UNOCHA. Overview (The Monthly Humanitarian Bulletin). (2018). Available online at: https://www.ochaopt.org/content/overview-november-2018. United Nations Office for the Coordination of Humanitarian Affairs. (Retrieved February 13, 2021).

[34] Abuzerr S Hadi M Zinszer K Nasseri S Yunesian M Mahvi AH. Comprehensive risk assessment of health-related hazardous events in the drinking water supply system from source to tap in gaza strip, palestine. J Environ Public Health. (2020) 48:1791–810. 10.1155/2020/7194780 32405304 PMC7204139

[35] Abuzerr S Nasseri S Yunesian M Hadi M Zinszer K Mahvi AH. Water, sanitation, and hygiene risk factors of acute diarrhea among children under five years in the Gaza Strip. J Water Sanit Hygiene Dev. (2020) 10:111–23. 10.2166/washdev.2019.072

[36] Abuzerr S Nasseri S Yunesian M Hadi M Mahvi AH Nabizadeh R. Prevalence of diarrheal illness and healthcare-seeking behavior by age-group and sex among the population of Gaza strip: a community-based cross-sectional study. BMC Public Health. (2019) 19:704. 10.1186/s12889-019-7070-0 31174512 PMC6555956

[37] Abuzerr S Nasseri S Yunesian M Yassin S Hadi M Mahvi AH. Microbiological quality of drinking water and prevalence of waterborne diseases in the Gaza strip, Palestine: a narrative review. J Geosci Environ Protect. (2019) 7:122. 10.4236/gep.2019.74008

[38] UNICEF. State of Palestine. WASH: water, sanitation and hygiene. (2020). Available online at: https://www.unicef.org/sop/whatwe-do/wash-water-sanitation-and-hygiene (Retrieved April 14, 2021).

[39] Abuzerr S Nasseri S Yunesian M Hadi M Mahvi AH Nabizadeh R. Household drinking water safety among the population of Gaza Strip, palestine: knowledge, attitudes, practices, and satisfaction. J Water Sanit Hygiene Dev. (2019) 9:500–12. 10.2166/washdev.2019.134

[40] Bank W. WASH (Water, Sanitation & Hygiene) COVID-19). (2020). Available online at: https://www.worldbank.org/en/topic/water/brief/wash-water-sanitation-hygiene-and-covid-19 (Retrieved March 1, 2021).

[41] UNOCHA. Waste Away: Living Next to a Dumpsite. Posted on 20 July 2020 as part of The Humanitarian Bulletin. (2020). Available online at: https://www.ochaopt.org/content/waste-away-living-next-dumpsite

[42] OCHA. COVID-19 Crisis. (2020). Available online at: https://www.ochaopt.org/covid-19

[43] Abuzerr S. The Impact of Environmental Pollution on Public Health in Light of the COVID-19 Pandemic in Fragile Conflict Settings: Reflections from the Gaza Strip. HEINRICH-BÖLL-STIFTUNG Palestine Jordan-PENGON-FOE Palestine. (2020). Available online at: https://ps.boell.org/en/2021/02/08/impact-environmental-pollution-public-health-light-covid-19-pandemic-fragile-and (Retrieved April 2, 2021).

[44] WHO. The Commission Calls for Closing the Health Gap in a Generation: Health Equity Through Action on the Social Determinants of Health. (2008). Available online at: https://www.who.int/social_determinants/final_report/csdh_finalreport_2008 (Retrieved February 17, 2021).

[45] Abuzerr S Nasseri S Yunesian M Hadi M Zinszer K Mahvi AH. Water, Sanitation, and Hygiene Risk Factors of Acute Diarrhea Among Under-Five Children in the Gaza Strip. Journal of Water, Sanitation and Hygiene for Development (2019).

[46] UNICEF. The Situation of Palestinian Children in the Occupied Palestinian Territory, Jordan, Syria and Lebanon. (2010). Available online at: https://www.unicef.org/oPt/PALESTINIAN_SITAN-final.pdf (Retrieved April 13, 2021).

